# The Relationship between Training and Mental Health among Caregivers of Individuals with Polytrauma

**DOI:** 10.1155/2015/185941

**Published:** 2015-12-07

**Authors:** Lillian Flores Stevens, Treven C. Pickett, Kathryn P. Wilder Schaaf, Brent C. Taylor, Amy Gravely, Courtney Harold Van Houtven, Greta Friedemann-Sánchez, Joan M. Griffin

**Affiliations:** ^1^Hunter Holmes McGuire VAMC, Psychology Section (116B), 1201 Broad Rock Boulevard, Richmond, VA 23249, USA; ^2^Departments of Psychology, Physical Medicine and Rehabilitation, Virginia Commonwealth University, Richmond, VA, USA; ^3^Departments of Psychology, Psychiatry, Physical Medicine and Rehabilitation, Virginia Commonwealth University, Richmond, VA, USA; ^4^Center for Chronic Disease Outcomes Research, Minneapolis VA Health Care System, 1 Veterans Drive (152/Building 9), Minneapolis, MN 55417, USA; ^5^Center for Health Services Research in Primary Care, Durham VA Medical Center, 508 Fulton Street, Durham, NC 27705, USA; ^6^Humphrey School of Public Affairs, University of Minnesota, 267 Humphrey Center, 301 19th Avenue S., Minneapolis, MN 55455, USA; ^7^Mayo Clinic, Department of Health Sciences Research, Division of Health Care Policy and Research, Kern Center for the Science of Health Care Delivery, 200 First Street SW, Rochester, MN 55905, USA

## Abstract

This was a hypothesis-generating exploration of relationships between caregiver training during TBI/polytrauma rehabilitation and caregiver mental health. In this cross-sectional study, 507 informal caregivers to US service members with TBI who received inpatient rehabilitation care in a Veterans Affairs' Polytrauma Rehabilitation Center from 2001 to 2009 completed a retrospective, self-report survey. Embedded in the survey were measures of caregiver mental health, including the National Institutes of Health's Patient Reported Outcome Measurement Information System (PROMIS) Anxiety and Depression Short Forms, the Rosenberg Self-Esteem scale, and the Zarit Burden Short Form. Though no groups endorsed clinical levels, mental health symptoms varied by caregiver training category (Trained, Not Trained, and Did Not Need Training). Caregivers who did not receive training on how to navigate healthcare systems endorsed higher depression and burden and lower self-esteem than those who did. Caregivers who did not receive training in supporting their care recipients' emotions endorsed higher anxiety, depression, and burden and lower self-esteem than those who did. Analyses also suggested a different association between training and mental health based on caregivers' relationship to the care recipient and the intensity of care recipient needs. Potential hypotheses for testing in future studies raised by these findings are discussed.

## 1. Introduction 

Traumatic brain injury (TBI) is considered the signature injury of the Operation Enduring Freedom and Operation Iraqi Freedom (OEF/OIF) conflicts [1]. Approximately 327,299 US military service members have been diagnosed with TBI between 2000 and the first quarter of 2015 [2]. Among the 25,044 service members injured in 2014 alone, 146 (0.6%) sustained a severe TBI, 2,010 (8.0%) sustained a moderate TBI, 20,972 (83.7%) sustained a mild TBI, and 1,759 (7.0%) sustained unclassifiable injuries [2]. Service members who sustain a TBI often sustain additional and potentially life-threatening traumatic injuries to other body systems and organs, including fractures, burns, hearing loss, vision loss, and amputation. This constellation of injuries is known as “polytraumatic injuries” [3, 4]. TBI with polytraumatic injuries are often accompanied by pain symptoms and a range of psychiatric comorbidities [3].

In 2005, the Department of Veterans Affairs designated a Polytrauma System of Care, which included Polytrauma Rehabilitation Centers (PRCs) that specialized in inpatient rehabilitation of TBI with polytrauma. The charge for the PRCs was to provide patient and family-centered inpatient care and life-long case management to those with polytrauma, especially those with moderate to severe war-related injuries. Patients present to the PRCs with mild to severe penetrating and nonpenetrating head injuries and have, on average, five injuries and six impairments. Nearly 90% have some cognitive impairment during their inpatient stay [4]. In the PRCs, the TBI defines the rehabilitation process, and therefore we refer to the constellation of injuries as TBI/polytrauma.

As TBI/polytrauma injuries result in a spectrum of behavioral, cognitive, emotional, and physical impairments [3, 5], injured individuals often require supervision and support from caregivers, usually parents or spouses [6]. Research within civilian populations indicates that TBI injuries impact caregiver psychosocial functioning, including financial and employment difficulty [7–9], marital strain [10], reductions in social activities [8, 9], poorer quality of life and perceived physical health [11, 12], higher prescription and nonprescription drug consumption [7–9], and higher levels of stress, anxiety, depression [13–16], and burden [8, 9, 13].

Despite the robust literature pointing to myriad biopsychosocial problems experienced by TBI caregivers in civilian healthcare settings, fewer studies have explored difficulties among caregivers of OEF/OIF service members with TBI/polytrauma injuries. Though there are similarities in caregiving experiences across TBI settings, adjustment to the caregiver role may be different between settings in several important ways. For example, as a prelude to the service members' injuries, caregivers of US service members may experience adjusting to the caregiving role after having experienced predeployment stress and ongoing stress and worry for the service members' well-being during deployment [17]. Further, the injury may signify not only a change in family roles, but also a potential loss of military identity, with transitions from military life to civilian life. Strong feelings of loss and abandonment can be part of the families' transition from military to civilian settings [17]. Lastly, most veterans who have experienced TBI also have comorbid psychiatric diagnoses, with PTSD being among the most common [18].

Comprehensively training caregivers to engage in learning specific skills may improve rehabilitation outcomes for patients who sustained a TBI, while simultaneously improving longer-term physical and mental health outcomes for the caregiver. Caregiver information and training are known to have positive effects on the outcomes of caregivers of individuals with dementia [19, 20] and to reduce anxiety and stress among caregivers of critically ill patients, thus facilitating coping [21]. Among caregivers of stroke patients, the provision of information appears to improve mental health outcomes [22]. Little is known about the potential benefits of targeted caregiver training in the OEF/OIF TBI/polytrauma context. Providing training and education to caregivers of injured service members who have sustained polytraumatic injuries may lead to outcomes similar to those found among caregivers of other conditions, especially TBI. However, with the constellation of injuries, the long-term visible (e.g., disfigurement, scaring, and vision loss) and invisible outcomes and comorbid conditions (e.g., PTSD, seizures, and attention difficulties), and the young age of the patients, the impact of training on caregiver outcomes may also be unique [3, 23]. Previous studies identified the effect of information provision to caregivers of TBI/polytrauma injured patients on their outcomes and questioned possible differential effects based on injury severity and the relationship to the patient [23, 24]. The current study on this unique population begins to fill this identified gap. The purpose of this hypothesis-generating study was to explore relationships between caregiver training and caregiver mental health outcomes in 507 family caregivers of US service members who sustained TBI/polytrauma injuries that necessitated inpatient rehabilitation care in one of four regional Polytrauma Rehabilitation Centers (PRCs).

## 2. Method

### 2.1. Participants

Study participants were the primary caregiver of patients identified in VA administrative records as having served during OEF/OIF; had polytrauma injuries or sequelae, including a TBI; received care and had been discharged for at least three months from one of four PRCs between September 2001 and February 2009; and were alive at the time the mailed survey was conducted [25]. This study explored characteristics of the 507 (out of 564) survey participants who responded to questions about two specific training needs.  Table 1 provides sample characteristics for the caregivers. Characteristics of this subsample of 507 are comparable to the larger population of 564 presented by Griffin and colleagues [25].

### 2.2. Data Collection

Study protocols, including waivers of documentation of informed consent, were approved by the Institutional Review Boards at all sites. The next of kin of all patients identified from VA administrative data who met inclusion criteria (*n* = 1, 045) were contacted. Data for each of these patients were also extracted from patient medical records. Initial survey packets included a cover letter, the questionnaire, a postage-paid return envelope, a $20 cash incentive, and a caregiver nomination form for the next of kin to complete in case the next of kin did not consider him/herself the primary caregiver. Multiple attempts were made by mail and telephone to reach nonrespondents.

Among the caregivers reached by mail or phone who could verify they were a caregiver we had a 67% response rate. Caregivers of those with lower functional status (measured by Functional Independence Measure (FIM) at both PRC admission and discharge) were significantly more likely to respond than caregivers of those with higher functional status. There were no significant differences between survey respondents and nonrespondents by care recipients' demographic characteristics, geographic location of injury (e.g., Iraq and US), or mechanism of injury (e.g., blast, motor vehicle crash, and fall).

### 2.3. Measures

The study questionnaire included questions about both the care recipient and caregiver. As part of the questionnaire, caregivers were asked if they had received training from a doctor, nurse, social worker, or some other health care provider on any of 11 specified tasks, including (1) navigating VA or Department of Defense (DoD) benefit or medical systems, (2) administering medications or helping with side effects, (3) changing bandages or dressings, (4) helping with pain, (5) helping with prosthetic devices or aids, (6) helping with assistive devices or aids, (7) helping with mobility devices or aids, (8) managing seizures, (9) supporting care recipients' emotions or feelings, (10) changing external catheter or colostomy bag, or (11) other training. This list of training tasks was developed using data from interviews with PRC clinical staff on caregiver needs [3] and refined with interviews conducted with caregivers. Additionally, these were tasks that may occur early on or intermittently during rehabilitation for polytrauma, depending on type and severity of injuries, as well as more universal needs that were not dependent on injury severity and could persist well past the rehabilitation period.

At the time of this survey, the PRCs were relatively new, the complexity of injuries was new, and the patient population was much younger than what the rehabilitation units were accustomed to managing [3]. Although there were efforts made at individual sites to provide high quality care and support to caregivers, there were no requirements for training caregivers and no uniform or manualized training for caregivers across PRCs. Efforts began in 2007 to develop a unified approach with formal training for caregivers across the four centers, including work on the Family Care Map [26, 27], which provided a collaborative approach for establishing standards of practice. A Polytrauma Family Education Manual was developed in 2007 that offered information about (1) the rehabilitation process, (2) background education on the medical, behavioral, and cognitive effects of TBI, and (3) caregiver education on dealing with difficult emotions such as anxiety, unrealistic expectations, frustration, stress, and depression after a loved one has been injured. Within this context, the training questions were intended to identify areas where future training programs could be developed, not to assess which training programs had been used or were most effective. Therefore, it is unknown if any training offered to or received by respondents was through the VA or through other resources, whether any standardized protocols were used among all four centers prior to 2007, or whether the manual was utilized in a standardized way across all four centers after 2007.

From the list of eleven training needs, this study focused on two of the more universal needs: (1) the need for training in navigating the VA or DoD benefits or medical system, and (2) the need for training in supporting their care recipient's emotions or feelings. These needs were chosen because previous research showed these were the top two needs with which caregivers of injured US Service Members reported needing help [3]. Response choices were “yes,” “no,” or “not needed.” For clarity we refer to these as “Trained,” “Not Trained,” and “Training Not Needed.” Notably, there could be overlap between caregivers who selected that they did not receive training and those who endorsed not needing the training.

In addition to training-specific questions, caregivers were asked to report demographic information about themselves and their care recipient, to describe the relationship between caregiver and care recipient, and to provide information about their care recipient's injuries. The intensity of care that caregivers provide was measured using caregiver report of care recipient's ability to perform activities of daily living (ADLs) [28], such as eating, bathing, and toileting; independent activities of daily living (IADLs) [29], such as managing money, doing chores, or cooking; or other forms of help and support, such as help with legal issues, navigating care systems, and managing pain or other symptoms. These activities were then coded into a variable with 3 categories: (1) assistance with one or more ADLs (high intensity), (2) assistance with one or more IADLs but no ADLs (moderate intensity), and (3) no assistance with any of the ADLs or IADLs (low intensity) [25].

FIM scores were aggregated from hospital discharge data. The FIM is a commonly used instrument in rehabilitation populations to assess motor and cognitive skills. Each item is scored 1 to 7. A score of 7 is categorized as “complete independence” and a score of 1 is “total assist” (performs less than 25% of task). Total functioning scores were calculated using both cognitive and motor skills.

Caregiving burden was assessed using the Zarit Burden Inventory (ZBI) scale (short-version), a 12-item, standardized, validated, reliable, and widely used measure of subjective burden associated with caregiving [30], including caregivers of those with TBI and PTSD [31, 32]. The ZBI uses a 5-point Likert scale ranging from “never” to “nearly always.” Generally, higher scores indicate higher burden. A score of 17 on the short form is considered the cutoff for high burden [30].

The National Institutes of Health's Patient Reported Outcome Measurement Information System (PROMIS) Anxiety and Depression Short Forms [33] were used to assess depression and anxiety symptoms. The eight-item depression scale and the seven-item anxiety scale both utilize five response categories (never, rarely, sometimes, often, and always). Scores were converted to a *T*-score metric based on a representative calibration sample consisting of the US general population and multiple disease populations [33, 34].

Rosenberg's Self-Esteem scale [35], a widely used assessment considered to be both reliable and valid with TBI populations [36], was used to assess caregiver self-esteem. The questionnaire consists of ten items with responses on a 4-point scale ranging from “strongly disagree” to “strongly agree” such that total scores range from 10 to 40, with higher scores indicating higher self-esteem [37].

### 2.4. Statistical Analyses

Statistical analyses were limited to descriptive statistics, given that descriptive analyses are more appropriate for hypothesis-generating studies, whereas inferential statistics are of more value for hypothesis-testing studies [38]. For each training task (i.e., navigating the VA/DoD; supporting emotions), descriptive statistics were compiled for all variables and means were compared, using *f*-tests from ANOVAs for continuous variables and chi-square tests for categorical variables, in order to determine if there were differences based on sociodemographics, injury and caregiver characteristics, and mental health outcomes between caregivers who received, did not receive, or did not need training.

Two stratified analyses were additionally conducted. First, considering the potential for relationship to the care recipient to impact caregivers' mental health, participants were separated into two relationship groups: parent and spouse/partner. The “other” group was excluded due to the inability to sensibly interpret any significant result based on the size of this group. Means were compared to explore if the three, nonoverlapping, training groups had different mental health outcomes within each relationship group. Second, because caregivers' mental health might be different based on the intensity of care recipient needs, participants were separated into “high” (requiring assistance with ADLs), “moderate” (needing help with IADLs), and “low” (not needing help with ADLs or IADLs) intensity of needs. Means were compared using ANOVA to explore if the three, nonoverlapping, training groups had different mental health outcomes within each intensity group.

We included caregivers who endorsed not needing training, speculating that their experiences were different from those who received or did not receive training. For example, it was possible that their burden was lower, their care recipients were potentially less likely to need help with ADLs or IADLs, they may have had previous care experiences, or they may have been trained in a medical field. Further, it is possible that there was overlap among response choices. For example, caregivers could have received training without needing it or could have needed training but not received it. Therefore, in order to reduce confusion or overlap with the “Training Not Needed” group, the above-described comparisons were also made between the “Trained” versus “Not Trained” groups only. Though results are described for ANOVAs that included the “Training Not Needed” group, given our interest in exploring the relationship between caregiver mental health and caregiver receipt of training, the interpretation of results is based on the “Trained” versus “Not Trained” group comparisons.

The 507 participants who responded to the two training questions are the maximum number of people available for analysis. However, for each question posed, the number available for analysis varies, due to responses for each training item. For example, a sample of the 507 might not have received the training, because they had no need for it. The number of people available for each question is clearly displayed in the results below as well as in each of the tables.

## 3. Results

Of the 564 study participants, 507 caregivers responded to the item regarding training for “Navigating the VA/DoD Benefits or Medical System.” The majority (*n* = 262, 51.7%) reported not receiving training, another 165 caregivers (32.5%) reported receiving training, and 80 caregivers (15.8%) reported not needing training. Likewise, 507 caregivers responded to the item regarding “Training for Supporting Care Recipient's Emotions or Feelings.” For this item, 226 caregivers (44.6%) reported receiving training, 215 (42.4%) reported not receiving training, and 66 caregivers (13.0%) reported not needing the training.

### 3.1. Navigating the VA/DoD

#### 3.1.1. Demographics, Injury, and Caregiver Characteristics (Table 2)

[Table tab2]The three training groups were similar in terms of age, gender, race, marital status, and education. The groups differed significantly by caregiver's relationship to care recipient. This difference did not remain significant when the Training Not Needed group was excluded from analysis. Among injury and caregiver characteristics, the groups differed only by intensity of care recipient needs, but again, this difference did not remain when the Not Needed group was excluded.

#### 3.1.2. Mental Health

Also shown in Table 2, caregivers in all three training groups endorsed anxiety and depression *T*-scores below 50, indicating that they were below the average compared to PROMIS measures' calibration sample consisting of the US general population and multiple disease populations [33, 34]. There were no differences in anxiety by training group. However, caregivers not trained in navigating the VA/DoD endorsed higher depression than those who received training or did not need training. Even after the Training Not Needed group was excluded from analyses, those who did not receive training had higher depressive symptoms than those who received training.

Caregivers among all three training groups endorsed high levels of self-esteem and there were no statistical differences among the groups. When the Training Not Needed group was excluded, those who received training had significantly higher self-esteem than those who did not receive training.

None of the three training groups endorsed clinically high levels of burden; however, burden was significantly higher among caregivers who did not receive training in navigating the VA/DoD compared to those who received training or did not need training. After the Training Not Needed group was excluded, caregivers who did not receive training endorsed higher burden than those trained.

Variation in mental health outcomes by caregivers' relationship to the care recipient was examined (Table 3). Among parents only, there was a significant difference in subjective burden by training, but this difference did not remain significant when the Training Not Needed group was excluded. Among spouses/partners, there were significant differences for the three training groups in anxiety, depression, and subjective burden, but only subjective burden remained significantly higher among those who did not receive training when the Training Not Needed group was excluded. Additionally, spouses/partners who did not receive training were the only participants who endorsed clinically significant levels of burden.

Variation in mental health outcomes by intensity of care recipient needs (i.e., high, moderate, and low intensity, as defined previously) was also examined (Table 4). Among the high intensity group, there were no significant differences in mental health among the training groups. Among the moderate intensity group, caregivers who did not need training reported lower burden than those who received training or did not receive training, but when the Not Needed group was excluded, there was no difference between caregivers who did or did not receive training. However, when the Training Not Needed group was excluded, two other significant differences emerged among the moderate intensity group; those who did not receive training endorsed higher depression and lower self-esteem than those who did receive training. Among the low intensity care group, caregivers who did not receive training endorsed significantly higher burden than those who received training or did not need training. Even when the Training Not Needed group was excluded, those who did not receive training still endorsed higher burden than those who received training. Additionally, caregivers of high intensity care recipients who did not receive training were the only participants who endorsed clinically significant levels of burden.

### 3.2. Supporting Emotions

#### 3.2.1. Demographics, Injury, and Caregiver Characteristics (Table 5)

[Table tab5]The three training groups were similar in terms of gender, marital status, and education. The three groups differed statistically on age, with the Training Not Needed group being a few years older than the Trained or Not Trained groups. However, there was no age difference among those who did or did not receive training when the Training Not Needed group was excluded. The three groups differed significantly regarding caregiver's relationship to care recipient, and this difference remained significant when the Training Not Needed group was excluded, with more parents receiving training and more spouses not receiving training. Race was not statistically different among the three groups but was significantly different when the Training Not Needed group was excluded. Among injury and caregiver characteristics, the three groups differed only on intensity of care recipient needs (the Training Not Needed group had fewer care recipients with high intensity needs), but there was no difference on intensity of needs between those who did and did not receive training when the Training Not Needed group was excluded.

#### 3.2.2. Mental Health

Caregivers in all three training groups yielded anxiety and depression *T* scores below 50, indicating that they were below the average compared to PROMIS measures' calibration sample consisting of the US general population and multiple disease populations [33, 34]. Caregivers who did not receive training in supporting their care recipient's feelings or emotions endorsed higher anxiety compared to those who did receive training or did not need training. Caregivers who did not receive training still endorsed higher anxiety than those who did when the Training Not Needed group was excluded. Similarly, those who were not trained in supporting emotions endorsed higher depression than those who did receive training or did not need the training and continued to endorse significantly higher depression when the Training Not Needed group was excluded.

Caregivers among all three training groups also endorsed high levels of self-esteem. There were no statistical differences among the three training groups, but when the Training Not Needed group was excluded, those who received training had statistically significantly higher self-esteem than those who did not receive training.

None of the three training groups endorsed clinically high levels of burden; however, burden was higher among caregivers who did not receive training in supporting emotions compared to those who did receive training or did not need training. Caregivers who did not receive training continued to endorse higher burden than those who did receive training even after the Training Not Needed group was excluded.

Variation in mental health outcomes by caregivers' relationship to the care recipient was also examined (Table 6). Among parents only, those who did not need training reported significantly lower burden than those who did or did not receive training, but there was no difference in burden between caregivers who did or did not receive training when the Training Not Needed group was excluded. Among spouses/partners, there were significant differences for the three training groups in depression and subjective burden. Those spouses/partners who did not receive training endorsed significantly higher depression and burden than those who did receive training, and those who did receive training endorsed higher depression and burden than those who did not need the training. Even after excluding the Training Not Needed group, caregivers who did not receive training endorsed higher depression and burden than those who did. Spouses/partners who did not receive training were the only participants who endorsed clinically significant levels of burden. Additionally, significant differences between the Trained and Not Trained groups arose for spouses in self-esteem; spouses who did not receive training endorsed lower self-esteem than those who did receive training.

Variation in mental health outcomes by intensity of care recipient needs (i.e., high, moderate, and low intensity, as defined previously) was also examined (Table 7). Among the high intensity group, there were no significant differences in mental health among the training groups. Caregivers from both the Trained and Training Not Received groups endorsed levels of burden right at the cutoff for clinical severity. Among the moderate intensity group, those who did not receive training in supporting emotions endorsed higher depression and burden than those who did not need the training or who did receive it. Even after excluding the Training Not Needed group, caregivers of care recipients with moderate intensity needs who did not receive training endorsed higher depression and burden than those who did receive training. Additionally, when the Training Not Needed group was excluded, two other significant differences emerged: caregivers of moderate intensity care recipients who did not receive training endorsed higher anxiety and lower self-esteem than those who did receive training. Among the low intensity group, though, there were no significant differences regarding anxiety, depression, or self-esteem. Caregivers who did not receive training endorsed higher burden than those who did receive training or did not need training and continued to endorse higher burden than those who did receive training after the Training Not Needed group was excluded.

## 4. Discussion

This exploratory study investigated relationships between training and mental health outcomes in a sample of family caregivers of US service members who sustained TBI/polytrauma injuries serious enough to necessitate acute inpatient rehabilitation care. Though none of the groups endorsed clinical levels of mental health symptomatology, caregivers who received training in how to navigate healthcare, benefits, and disability systems endorsed lower depression, lower burden, and higher self-esteem than those who did not. Additionally, caregivers who received training in supporting the care recipient's emotions reported lower anxiety, depression, and burden and higher self-esteem than those who did not. These exploratory findings are unique in the polytrauma literature, raise hypotheses for future testing, and point to the potential for fruitful clinical research interventions.

A recent study found that emotional and instrumental support are the most frequently unmet needs among caregivers of injured patients receiving care at PRCs [39]. Previous research among caregivers of individuals with TBI provides evidence that spouses or partners experience higher stress levels, role changes, and health issues than parents [40, 41]. Similarly, our results suggest that the association between caregivers' training and mental health could differ based on relationship to the care recipient. For parents, receiving or not receiving training was not associated with mental health, but spouses who received training reported better mental health than spouses who did not receive training. This finding raises several hypotheses. The fact that training was not associated with mental health for parents may reflect parents' innate caregiving approach to their children and the relative ease they have in reverting back to previously held parenting roles. Training to emotionally support a child may not be as relevant for parents. In contrast, spouses may feel as though they have lost their partner, or that their partner has profoundly changed and, consequently, may undergo a more significant shift in roles when they become caregivers. They, therefore, may benefit more from such training. Future studies should test the hypotheses that caregiver needs differ by relationship and elucidate the underlying reasons.

Studies among caregivers of patients with dementia have shown that training programs aimed at improving management and emotional skills have positive effects on the health of caregivers [19, 20]. Similarly, this study suggests there is an association between caregivers' training and mental health and that these benefits varied by the intensity of care needed by care recipients. With no association observed between training and mental health among caregivers of care recipients with high intensity needs, we suggest further research to test if training is not as beneficial because caregivers of patients with ADL dependence often have skilled, professional care to help and provide respite or if lessons from training are not easily absorbed because of the physical, emotional, and spiritual burden of care. Future research is needed to confirm whether the intensity of the care recipient's needs and their physical, cognitive, and behavioral functioning influence the mental health outcomes of caregivers and how training may benefit caregiver mental health outcomes and elucidate potential explanations. Furthermore, future research may provide helpful guidance to interdisciplinary teams as to whether the intensity of caregiver training should correspond with the severity of patient injuries or if targeted, specific training is equally important for caregivers of those with moderate- or mild-severity TBI injuries.

Multiple training and educational opportunities are now available through the Veteran's Affairs (VA) Polytrauma System of Care that was not available when these data were collected. A 2010 VA policy, in fact, mandates that therapists and providers document efforts to prepare family members for changes associated with severe injury [42]. Completion of caregiver training is also now a requirement for caregiver benefits authorized through the Caregivers and Veterans Omnibus Health Service Act of 2010 [43]. Benefits offered through the VA's Comprehensive Caregiver Program provide financial stipends, health care, respite, counseling, and travel reimbursement to eligible caregivers of veterans injured in OEF/OIF. No empirical studies have yet examined whether the training interventions lead to carryover of caregiver skills into real-world settings, or whether family members transitioning from an inpatient rehabilitation environment feel prepared for the realities of community reintegration. Given the heterogeneity of TBI/polytrauma clinical presentations, current rehabilitation interventions are highly individualized for both TBI and family systems, which impedes opportunities for more robust, longitudinal investigations on the effectiveness of any one targeted educational intervention for TBI/polytrauma caregivers. Future research could develop, implement, and test feasibility, efficacy, and effectiveness of caregiver training approaches that have shared basic foundational elements while also leaving room for individualized skill training. Future studies can also test the timing of caregiver interventions, called for in previous studies [23, 24], throughout the transitions from inpatient to outpatient care to optimize learning and carryover of important caregiver skills, especially for those caregivers who report negative longer term mental health outcomes.

Although this study's findings elicit important hypotheses and suggestions for future research, a number of limitations related to study design need to be acknowledged. First, this was an exploratory, hypothesis-generating, and cross-sectional study. Analyses were limited to descriptive statistics [38] and, as the mean comparisons via ANOVA and chi-square were meant to be exploratory, no effect sizes or equality of variances were reported. Further, the analyses did not control for variables that may complicate caregiver roles, such as mental health history of PTSD, depression, or substance abuse. Second, the survey questions were retrospective. Though there is strength in exploring caregivers' perception of training experiences, family members/caregivers of patients in the acute phase of rehabilitation may be in states of emotional distress that render their later recollection of caregiver training unreliable. Third, the survey did not capture sources and types of training offered. With no standardized training protocols existing at the four PRCs during the time of data collection, it is unknown exactly what kind of training was offered by each of the four PRC sites. Fourth, it is possible that there was overlap among response choices for the training questions, in that caregivers could have received training without needing it or could have needed training but not received it. Finally, no caregivers for service members who received PRC care after 2009 were included; results may not generalize to families with service members who were injured after 2009. Available education and training developments across PRCs (plus the addition of a fifth PRC in San Antonio) could not have been taken into account in this study. Results therefore should be considered preliminary and informative for possible future research, as no strong causal interpretations about training can be made from these cross-sectional associations.

## 5. Conclusions

Though preliminary, results of this study suggest that caregiver training in navigating their care recipient's systems of healthcare and disability, or on how to support their care recipient's emotions, is positively associated with mental health outcomes among caregivers for OEF/OIF service members who have sustained TBI/polytrauma necessitating inpatient rehabilitation. Findings raise several hypotheses; first, caregiver needs may differ by relationship. Spouses/partners of individuals with TBI/polytraumatic injuries may have higher levels of emotional stress than parents, consistent with other literature. Second, training may not be as beneficial for caregivers of individuals with ADL dependence. This may be because they often have skilled, professional care to help and provide respite, or because these caregivers have physical, emotional, and spiritual burdens of care that impede learning or benefit from training. Future work should more rigorously test these hypotheses, as well as investigate the possibility of standardized caregiver training interventions that maintain clinical sensitivity for relationship to care recipient and intensity of care recipient's needs, while also allowing the interventions to be assessed empirically for efficacy and effectiveness.

## Figures and Tables

**Table 1:** Sample characteristics.

	*N* ^*∗*^	Caregivers
CG age (*x*, SD)	482	47.16 (12.56)
CG gender (count, %)	495	—
Male	—	95 (19%)
Female	—	400 (81%)
CG race (count, %)	507	—
White	—	375 (74%)
Black	—	43 (9%)
More than 1	—	17 (3%)
Other	—	27 (5%)
Unknown	—	45 (9%)
CG marital status (count, %)	495	—
Married	—	362 (73%)
Divorced	—	63 (13%)
Living with partner	—	19 (4%)
Separated	—	15 (3%)
Widowed	—	22 (4%)
Never married	—	14 (3%)
CG education (count, %)	494	—
Less than HS	—	11 (2%)
Some HS	—	15 (3%)
HS graduate/GED	—	103 (21%)
Vocational school	—	34 (7%)
Some college	—	138 (28%)
Associate's degree	—	57 (11%)
College degree	—	88 (18%)
M.S. or doctoral degree	—	48 (10%)
CG relationship to CR	507	—
Parent	—	302 (60%)
Spouse/partner	—	174 (34%)
Other	—	31 (6%)
CR months since injury (*x*, SD)	470	51.31 (24.13)
Months of caregiving (*x*, SD)	471	45.97 (23.65)

^*∗*^The *N* varied for some of these variables due to missing or incomplete data for these items.

Note: 507 of the total 564 caregivers responded to the item regarding training for navigating the VA or DOD Benefits or Medical System. CG: caregiver; CR: care recipient.

**Table 2 tab2:** Comparison of navigating VA or DoD training groups on sociodemographics, injury and caregiver characteristics, and mental health outcomes.

	*N*	Training (*n* = 507)			Significantly different?	
		Trained	Not Trained	Training Not Needed	Trained versus Not Trained versus Training Not Needed	Trained versus Not Trained
CG age (*x*, SD)	482	48.22 (12.00)	46.51 (12.78)	47.00 (13.01)	*p* = .40 (*N* = 482)	*p* = .17 (*N* = 410)
CG gender (count, %)	495	—	—	—	*p* = .54 (*N* = 495)	*p* = .19 (*N* = 420)
Male	—	36 (22%)	46 (18%)	13 (17%)	—	—
Female	—	128 (78%)	210 (82%)	62 (83%)	—	—
CG race (count, %)	507	—	—	—	*p* = .77 (*N* = 507)	*p* = .69 (*N* = 427)
White	—	126 (76%)	194 (74%)	55 (69%)	—	—
Black	—	12 (7%)	25 (10%)	6 (7%)	—	—
More than 1	—	6 (4%)	8 (3%)	3 (4%)	—	—
Other	—	10 (6%)	11 (4%)	6 (7%)	—	—
Unknown	—	11 (7%)	24 (9%)	10 (13%)	—	—
CG marital status (count, %)	495	—	—	—	*p* = .33 (*N* = 495)	*p* = .20 (*N* = 420)
Married	—	122 (74%)	189 (74%)	51 (68%)	—	—
Divorced	—	19 (12%)	32 (13%)	12 (16%)	—	—
Living with partner	—	3 (2%)	11 (4%)	5 (7%)	—	—
Separated	—	8 (5%)	6 (2%)	1 (1%)	—	—
Widowed	—	10 (6%)	9 (3.5%)	3 (4%)	—	—
Never married	—	2 (1%)	9 (3.5%)	3 (4%)	—	—
CG education (count, %)	494	—	—	—	*p* = .16 (*N* = 494)	*p* = .19 (*N* = 419)
Less than HS	—	3 (2%)	6 (2%)	2 (3%)	—	—
Some HS	—	6 (4%)	7 (3%)	2 (3%)	—	—
HS graduate/GED	—	33 (20%)	52 (20%)	18 (24%)	—	—
Vocational school	—	9 (5%)	15 (6%)	10 (13%)	—	—
Some college	—	51 (31%)	71 (28%)	16 (21%)	—	—
Associate's degree	—	9 (5%)	37 (15%)	11 (15%)	—	—
College degree	—	36 (22%)	41 (16%)	11 (15%)	—	—
M.S. or doctoral degree	—	17 (11%)	26 (10%)	5 (6%)	—	—
CG relationship to CR	507	—	—	—	*p* = .01 (*N* = 507)	*p* = .08 (*N* = 427)
Parent	—	108 (65%)	145 (55%)	49 (61%)	—	—
Spouse/partner	—	52 (32%)	101 (39%)	21 (26%)	—	—
Other	—	5 (3%)	16 (6)	10 (13%)	—	—
CR months since injury (*x*, SD)	470	48.36 (22.41)	52.28 (23.89)	54.75 (28.09)	*p* = .12 (*N* = 470)	*p* = .10 (*N* = 400)
Months of caregiving (*x*, SD)	471	44.82 (22.04)	47.34 (23.39)	43.68 (27.75)	*p* = .40 (*N* = 471)	*p* = .28 (*N* = 401)
CR total FIM (*x*, SD)	497	104.68 (28.33)	109.40 (24.04)	116.87 (15.56)	*p* = .00 (*N* = 497)	*p* = .07 (*N* = 418)
Intensity of CR needs (count, %)	507	—	—	—	*p* = .00 (*N* = 507)	*p* = .72 (*N* = 427)
ADLs+	—	40 (24%)	72 (27%)	6 (8%)	—	—
Only IADs+	—	90 (55%)	140 (54%)	33 (41%)	—	—
No help needed with I/ADLs	—	35 (21%)	50 (19%)	41 (51%)	—	—
CR injury severity (count, %)	425	—	—	—	*p* = .19 (*N* = 425)	*p* = .19 (*N* = 360)
Fully conscious	—	13 (9%)	21 (9%)	4 (6%)	—	—
Unconscious ≤ 30 min	—	15 (11%)	42 (19%)	9 (14%)	—	—
Unconscious ≥ 30 min, ≤1 week	—	33 (24%)	50 (23%)	22 (34%)	—	—
Unconscious ≥ 1 week	—	79 (56%)	107 (49%)	30 (46%)	—	—
CG anxiety	489	48.49 (9.21)	49.73 (10.94)	47.48 (11.73)	*p* = .22 (*N* = 489)	*p* = .23 (*N* = 418)
CG depression	489	46.92 (9.36)	49.79 (9.87)	47.14 (11.54)	*p* = .01 (*N* = 489)	*p* = .00 (*N* = 418)
CG self-esteem	486	33.65 (4.74)	32.43 (5.95)	33.40 (5.58)	*p* = .07 (*N* = 486)	*p* = .03 (*N* = 416)
CG subjective burden	495	12.17 (9.73)	14.49 (10.32)	5.67 (7.65)	*p* = .00 (*N* = 495)	*p* = .02 (*N* = 420)

Note: percentages based on column total; ADLs: activities of daily living; CG: caregiver; CR: care recipient; FIM: Functional Independence Measure; IADLs: instrumental activities of daily living; Min: minutes.

**Table 3:** Differences in caregiver mental health based on training received for navigating the VA or DoD based on caregivers' relationship to care recipient.

	*N*	Training			Significantly different?	
		Trained	Not Trained	Training Not Needed	Trained versus Not Trained versus Training Not Needed	Trained versus Not Trained
Parents (*n* = 302)						
CG anxiety	287	46.95 (8.15)	47.19 (10.06)	48.94 (13.04)	*p* = .53 (*N* = 287)	*p* = .84 (*N* = 247)
CG depression	287	45.74 (8.49)	47.90 (9.39)	49.27 (12.83)	*p* = .08 (*N* = 287)	*p* = .06 (*N* = 247)
CG self-esteem	286	34.30 (4.52)	33.49 (5.23)	33.15 (6.00)	*p* = .34 (*N* = 286)	*p* = .21 (*N* = 246)
CG subjective burden	291	11.05 (8.91)	12.11 (9.64)	5.59 (7.39)	*p* = .00 (*N* = 291)	*p* = .38 (*N* = 247)

Spouses/partners (*n* = 174)						
CG anxiety	171	52.47 (10.22)	53.21 (10.70)	46.07 (10.14)	*p* = .02 (*N* = 171)	*p* = .69 (*N* = 150)
CG depression	171	49.98 (10.58)	52.79 (9.95)	45.55 (9.15)	*p* = .02 (*N* = 171)	*p* = .11 (*N* = 150)
CG self-esteem	170	32.36 (5.13)	30.69 (6.57)	33.21 (5.24)	*p* = .11 (*N* = 170)	*p* = .12 (*N* = 150)
CG subjective burden	174	14.59 (10.97)	18.31 (10.34)	5.71 (8.49)	*p* = .00 (*N* = 174)	*p* = .04 (*N* = 153)

Note: CG: caregiver.

**Table 4:** Differences in caregiver mental health based on training received for navigating the VA or DoD based on intensity of care recipient's needs.

	*N*	Training			Significantly different?	
		Trained	Not Trained	Training Not Needed	Trained versus Not Trained versus Training Not Needed	Trained versus Not Trained
High intensity care recipient needs (ADLS+) (*n* = 118)						
CG anxiety	117	50.93 (10.29)	53.05 (10.64)	44.98 (10.43)	*p* = .16 (*N* = 117)	*p* = .31 (*N* = 111)
CG depression	117	50.83 (9.84)	52.76 (8.30)	46.90 (7.21)	*p* = .21 (*N* = 117)	*p* = .27 (*N* = 111)
CG self-esteem	114	32.78 (5.12)	31.49 (6.35)	35.02 (4.77)	*p* = .29 (*N* = 114)	*p* = .28 (*N* = 109)
CG subjective burden	115	15.94 (9.23)	18.54 (10.27)	12.20 (11.73)	*p* = .21 (*N* = 115)	*p* = .19 (*N* = 110)

Moderate intensity care recipient needs (only IADLs+) (*n* = 263)						
CG anxiety	257	48.73 (9.05)	50.10 (10.73)	50.25 (12.45)	*p* = .59 (*N* = 257)	*p* = .32 (*N* = 224)
CG depression	257	46.64 (9.20)	49.79 (10.38)	48.46 (12.67)	*p* = .08 (*N* = 257)	*p* = .02 (*N* = 224)
CG self-esteem	255	33.99 (4.54)	32.51 (5.85)	32.34 (5.22)	*p* = .10 (*N* = 255)	*p* = .04 (*N* = 223)
CG subjective burden	262	13.55 (9.50)	14.60 (9.49)	7.85 (8.52)	*p* = .00 (*N* = 262)	*p* = .42 (*N* = 229)

Low intensity care recipient needs (no help needed) (*n* = 126)						
CG anxiety	115	45.05 (7.35)	43.76 (9.67)	45.10 (10.86)	*p* = .76 (*N* = 115)	*p* = .51 (*N* = 83)
CG depression	115	43.20 (7.59)	45.40 (9.03)	45.83 (11.09)	*p* = .45 (*N* = 115)	*p* = .25 (*N* = 83)
CG self-esteem	117	33.80 (4.83)	33.55 (5.52)	34.18 (5.97)	*p* = .88 (*N* = 117)	*p* = .83 (*N* = 84)
CG subjective burden	118	3.59 (5.02)	8.38 (9.90)	2.84 (4.68)	*p* = .00 (*N* = 118)	*p* = .01 (*N* = 81)

Note: ADLs: activities of daily living; CG: caregiver; CR: care recipient; IADLs: instrumental activities of daily living.

**Table 5 tab5:** Comparison of supporting emotions training groups on sociodemographics, injury and caregiver characteristics, and mental health outcomes.

	*N*	Training (*n* = 507)			Significantly different?	
		Trained	Not Trained	Training Not Needed	Trained versus Not Trained versus Training Not Needed	Trained versus Not Trained
CG age (*x*, SD)	482	46.77 (12.14)	46.30 (13.32)	51.14 (11.55)	*p* = .03 (*N* = 482)	*p* = .70 (*N* = 424)
CG gender (count, %)	495	—	—	—	*p* = .58 (*N* = 495)	*p* = .22 (*N* = 432)
Male	—	36 (16%)	41 (20%)	13 (21%)	—	—
Female	—	186 (84%)	169 (80%)	50 (79%)	—	—
CG race (count, %)	507	—	—	—	*p* = .06 (*N* = 507)	*p* = .03 (*N* = 441)
White	—	169 (75%)	161 (75%)	47 (71%)	—	—
Black	—	19 (8%)	19 (9%)	4 (6%)	—	—
More than 1	—	10 (4%)	6 (3%)	1 (2%)	—	—
Other	—	17 (8%)	6 (3%)	4 (6%)	—	—
Unknown	—	11 (5%)	23 (10%)	10 (15%)	—	—
CG marital status (count, %)	495	—	—	—	*p* = .24 (*N* = 495)	*p* = .11 (*N* = 433)
Married	—	162 (72%)	156 (75%)	45 (73%)	—	—
Divorced	—	26 (11%)	29 (13%)	8 (13%)	—	—
Living with partner	—	6 (3%)	10 (5%)	2 (3%)	—	—
Separated	—	11 (5%)	3 (1%)	0 (0%)	—	—
Widowed	—	13 (6%)	5 (2%)	4 (6%)	—	—
Never married	—	6 (3%)	6 (3%)	3 (5%)	—	—
CG education (count, %)	494	—	—	—	*p* = .69 (*N* = 494)	*p* = .94 (*N* = 432)
Less than HS	—	3 (1%)	4 (2%)	4 (6%)	—	—
Some HS	—	8 (4%)	5 (2%)	2 (4%)	—	—
HS graduate/GED	—	46 (20%)	41 (20%)	15 (24%)	—	—
Vocational school	—	15 (7%)	14 (7%)	5 (8%)	—	—
Some college	—	67 (30%)	57 (27%)	13 (21%)	—	—
Associate's degree	—	26 (12%)	25 (12%)	5 (8%)	—	—
College degree	—	40 (18%)	37 (18%)	13 (21%)	—	—
M.S. or doctoral degree	—	19 (8%)	25 (12%)	5 (8%)	—	—
CG relationship to CR	507	—	—	—	*p* = .01 (*N* = 507)	*p* = .03 (*N* = 441)
Parent	—	143 (63%)	111 (52%)	47 (71%)	—	—
Spouse/partner	—	70 (31%)	92 (42%)	13 (20%)	—	—
Other	—	13 (6%)	12 (6%)	6 (9%)	—	—
CR months since injury (*x*, SD)	470	51.06 (23.44)	50.90 (23.60)	53.05 (28.00)	*p* = .83 (*N* = 470)	*p* = .95 (*N* = 411)
Months of caregiving (*x*, SD)	470	46.35 (22.96)	45.47 (23.23)	45.91 (27.23)	*p* = .93 (*N* = 470)	*p* = .70 (*N* = 412)
CR total FIM (*x*, SD)	496	107.74 (26.06)	109.92 (23.65)	113.23 (18.64)	*p* = .25 (*N* = 496)	*p* = .37 (*N* = 431)
Intensity of CR needs (count, %)	507	—	—	—	*p* = .00 (*N* = 507)	*p* = .12 (*N* = 441)
ADLs+	—	51 (23%)	60 (30%)	5 (8%)	—	—
Only IADs+	—	118 (52%)	117 (53%)	30 (45%)	—	—
No help needed with I/ADLs	—	57 (25%)	38 (17%)	31 (47%)	—	—
CR injury severity (count, %)	425	—	—	—	*p* = .31 (*N* = 425)	*p* = .37 (*N* = 374)
Fully conscious	—	14 (7%)	17 (9%)	7 (14%)	—	—
Unconscious ≤ 30 min	—	28 (14%)	35 (20%)	4 (8%)	—	—
Unconscious ≥ 30 min, ≤1 week	—	49 (25%)	45 (25%)	13 (25%)	—	—
Unconscious ≥ 1 week	—	104 (54%)	82 (46%)	27 (53%)	—	—
CG anxiety	489	47.92 (10.12)	50.34 (10.63)	47.04 (11.12)	*p* = .02 (*N* = 489)	*p* = .02 (*N* = 431)
CG depression	489	47.10 (9.31)	50.05 (10.26)	46.64 (10.91)	*p* = .00 (*N* = 489)	*p* = .00 (*N* = 431)
CG self-esteem	487	33.56 (5.02)	32.32 (5.91)	33.52 (5.64)	*p* = .05 (*N* = 487)	*p* = .02 (*N* = 428)
CG subjective burden	496	11.56 (9.16)	15.02 (10.86)	5.33 (10.19)	*p* = .00 (*N* = 496)	*p* = .00 (*N* = 435)

Note: Percentages based on column total. ADLs: activities of daily living; CG: caregiver; CR: care recipient; FIM: Functional Independence Measure; IADLs: instrumental activities of daily living; Min: minutes.

**Table 6:** Differences in caregiver mental health based on training received for supporting emotions based on relationship to care recipient.

	*N*	Training			Significantly different?	
		Trained	Not Trained	Training Not Needed	Trained versus Not Trained versus Training Not Needed	Trained versus Not Trained
Parents (*n* = 301)						
CG anxiety	286	46.59 (9.23)	47.70 (9.86)	47.26 (11.85)	*p* = .68 (*N* = 286)	*p* = .37 (*N* = 247)
CG depression	286	45.93 (8.74)	48.23 (9.98)	47.80 (11.33)	*p* = .15 (*N* = 286)	*p* = .06 (*N* = 247)
CG self-esteem	285	34.05 (4.80)	33.58 (5.13)	33.59 (5.75)	*p* = .74 (*N* = 285)	*p* = .46 (*N* = 245)
CG subjective burden	290	10.60 (8.60)	12.21 (10.15)	5.53 (6.84)	*p* = .00 (*N* = 290)	*p* = .18 (*N* = 248)

Spouses/partners (*n* = 175)						
CG anxiety	172	50.76 (10.67)	53.70 (10.61)	48.55 (9.60)	*p* = .10 (*N* = 172)	*p* = .09 (*N* = 159)
CG depression	172	49.60 (9.96)	53.11 (10.01)	44.99 (10.60)	*p* = .01 (*N* = 172)	*p* = .03 (*N* = 159)
CG self-esteem	171	32.56 (5.56)	30.50 (6.35)	32.77 (5.76)	*p* = .08 (*N* = 171)	*p* = .03 (*N* = 158)
CG subjective burden	175	13.53 (9.92)	18.94 (10.78)	4.77 (8.97)	*p* = .00 (*N* = 175)	*p* = .00 (*N* = 162)

Note: CG: caregiver; CR: care recipient.

**Table 7:** Differences in caregiver mental health based on training received for supporting emotions based on intensity of care recipient's needs.

	*N*	Training			Significantly different?	
		Trained	Not Trained	Training Not Needed	Trained versus Not Trained versus Training Not Needed	Trained versus Not Trained
High intensity care recipient needs (ADLS+) (*n* = 116)						
CG anxiety	115	51.12 (11.11)	52.70 (10.14)	46.22 (9.50)	*p* = .37 (*N* = 115)	*p* = .44 (*N* = 110)
CG depression	115	50.92 (9.26)	52.34 (8.54)	49.10 (7.96)	*p* = .58 (*N* = 115)	*p* = .41 (*N* = 110)
CG self-esteem	113	33.14 (5.30)	31.29 (6.23)	34.80 (5.59)	*p* = .16 (*N* = 113)	*p* = .10 (*N* = 108)
CG subjective burden	114	17.05 (9.23)	17.85 (10.44)	9.25 (10.69)	*p* = .25 (*N* = 114)	*p* = .67 (*N* = 110)

Moderate intensity care recipient needs (only IADLs+) (*n* = 265)						
CG anxiety	259	48.12 (9.85)	51.04 (10.38)	49.19 (12.24)	*p* = .10 (*N* = 259)	*p* = .03 (*N* = 229)
CG depression	259	46.63 (9.09)	50.40 (10.83)	48.32 (12.13)	*p* = .02 (*N* = 259)	*p* = .01 (*N* = 229)
CG self-esteem	257	33.68 (4.90)	32.18 (5.73)	33.22 (5.51)	*p* = .11 (*N* = 257)	*p* = .04 (*N* = 227)
CG subjective burden	264	12.28 (8.40)	15.57 (10.49)	7.97 (8.27)	*p* = .00 (*N* = 264)	*p* = .01 (*N* = 234)

Low intensity care recipient needs (no help needed) (*n* = 126)						
CG anxiety	115	44.53 (8.78)	44.44 (10.25)	44.43 (9.66)	*p* = .99 (*N* = 115)	*p* = .96 (*N* = 92)
CG depression	115	44.55 (8.87)	45.32 (9.71)	43.92 (9.48)	*p* = .84 (*N* = 115)	*p* = .70 (*N* = 92)
CG self-esteem	117	33.70 (5.06)	34.32 (5.57)	33.62 (5.99)	*p* = .83 (*N* = 117)	*p* = .58 (*N* = 93)
CG subjective burden	118	4.80 (6.23)	8.77 (10.49)	1.81 (3.06)	*p* = .00 (*N* = 118)	*p* = .03 (*N* = 91)

Note: ADLs: activities of daily living; CG: caregiver; CR: care recipient; IADLs: instrumental activities of daily living.

## References

[1] Okie S. (2005). Traumatic brain injury in the war zone. *The New England Journal of Medicine*.

[2] Defense and Veterans Brain Injury Center http://dvbic.dcoe.mil/dod-worldwide-numbers-tbi.

[3] Friedemann-Sánchez G., Sayer N. A., Pickett T. (2008). Provider perspectives on the rehabilitation of patients with polytrauma. *Archives of Physical Medicine and Rehabilitation*.

[4] Sayer N. A., Chiros C. E., Sigford B. (2008). Characteristics and rehabilitation outcomes among patients with blast and other injuries sustained during the global war on terror. *Archives of Physical Medicine and Rehabilitation*.

[5] Cavallo M. M., Kay T., Silver J. M., McAllister T. W., Yudofsky S. C. (2011). The family system. *Textbook of Traumatic Brain Injury*.

[6] Knight R. G., Devereux R., Godfrey H. P. D. (1998). Caring for a family member with a traumatic brain injury. *Brain Injury*.

[7] Hall K. M., Karzmark P., Stevens M., Englander J., O'Hare P., Wright J. (1994). Family stressors in traumatic brain injury: a two-year follow-up. *Archives of Physical Medicine and Rehabilitation*.

[8] Marsh N. V., Kersel D. A., Havill J. H., Sleigh J. W. (1998). Caregiver burden at 6 months following severe traumatic brain injury. *Brain Injury*.

[9] Marsh N. V., Kersel D. A., Havill J. H., Sleigh J. W. (1998). Caregiver burden at 1 year following severe traumatic brain injury. *Brain Injury*.

[10] Peters L. C., Stambrook M., Moore A. D., Esses L. (1990). Psychosocial sequelae of closed head injury: effects on the marital relationship. *Brain Injury*.

[11] McPherson K. M., Pentland B., McNaughton H. K. (2000). Brain injury—the perceived health of carers. *Disability and Rehabilitation*.

[12] Moules S., Chandler B. J. (1999). A study of the health and social needs of carers of traumatically brain injured individuals served by one community rehabilitation team. *Brain Injury*.

[13] Kreutzer J. S., Gervasio A. H., Camplair P. S. (1994). Patient correlates of caregivers' distress and family functioning after traumatic brain injury. *Brain Injury*.

[14] Marsh N. V., Kersel D. A., Havill J. H., Sleigh J. W. (2002). Caregiver burden during the year following severe traumatic brain injury. *Journal of Clinical and Experimental Neuropsychology*.

[15] Nabors N. A., Seacat J., Rosenthal M. (2002). Predictors of caregiver burden following traumatic brain injury. *Brain Injury*.

[16] Ponsford J., Olver J., Ponsford M., Nelms R. (2003). Long-term adjustment of families following traumatic brain injury where comprehensive rehabilitation has been provided. *Brain Injury*.

[17] Collins R. C., Kennedy M. C. (2008). Serving families who have served: providing family therapy and support in interdisciplinary polytrauma rehabilitation. *Journal of Clinical Psychology*.

[18] Taylor B. C., Hagel E. M., Carlson K. F. (2012). Prevalence and costs of co-occurring traumatic brain injury with and without psychiatric disturbance and pain among Afghanistan and Iraq war veteran VA users. *Medical Care*.

[19] Gottlieb B. H., Rooney J. A. (2004). Coping effectiveness: determinants and relevance to the mental health and affect of family caregivers of persons with dementia. *Aging and Mental Health*.

[20] Coon D. W., Rubert M., Solano N. (2004). Well-being, appraisal, and coping in Latina and Caucasian female dementia caregivers: findings from the REACH Study. *Aging and Mental Health*.

[21] Chiu Y.-L., Chien W.-T., Lam L.-W. (2004). Effectiveness of a needs-based education programme for families with a critically ill relative in an intensive care unit. *Journal of Clinical Nursing*.

[22] Perrin P. B., Johnston A., Vogel B. (2010). A culturally sensitive transition assistance program for stroke caregivers: examining caregiver mental health and stroke rehabilitation. *Journal of Rehabilitation Research and Development*.

[23] Griffin J. M., Friedemann-Sánchez G., Hall C., Phelan S., Van Ryn M. (2009). Families of patients with polytrauma: understanding the evidence and charting a new research agenda. *Journal of Rehabilitation Research and Development*.

[24] Friedemann-Sánchez G., Griffin J. M., Rettmann N. A., Rittman M., Partin M. R. (2008). Communicating information to families of polytrauma patients: a narrative literature review. *Rehabilitation Nursing*.

[25] Griffin J. M., Friedemann-Sánchez G., Jensen A. C. (2012). The invisible side of war: families caring for US service members with traumatic brain injuries and polytrauma. *Journal of Head Trauma Rehabilitation*.

[26] Hall C., Sigford B., Sayer N. (2010). Practice changes associated with the Department of Veterans Affairs' family care collaborative. *Journal of General Internal Medicine*.

[27] Ford J. H., Wise M., Krahn D., Oliver K. A., Hall C., Sayer N. (2014). Family care map: sustaining family-centered care in polytrauma rehabilitation centers. *Journal of Rehabilitation Research and Development*.

[28] Katz S., Ford A. B., Moskowitz R. W., Jackson B. A., Jaffe M. W. (1963). Studies of illness in the aged: the index of ADL: a standardized measure of biological and psychosocial function. *The Journal of the American Medical Association*.

[29] Lawton M. P., Brody E. M. (1969). Assessment of older people: self-maintaining and instrumental activities of daily living. *Gerontologist*.

[30] Bédard M., Molloy D. W., Squire L., Dubois S., Lever J. A., O'Donnell M. (2001). The Zarit Burden Interview: a new short version and screening version. *Gerontologist*.

[31] Calhoun P. S., Beckham J. C., Bosworth H. B. (2002). Caregiver burden and psychological distress in partners of veterans with chronic posttraumatic stress disorder. *Journal of Traumatic Stress*.

[32] Siegert R. J., Jackson D. M., Tennant A., Turner-Stokes L. (2010). Factor analysis and Rasch analysis of the Zarit burden interview for acquired brain injury carer research. *Journal of Rehabilitation Medicine*.

[33] Pilkonis P. A., Choi S. W., Reise S. P., Stover A. M., Riley W. T., Cella D. (2011). Item banks for measuring emotional distress from the patient-reported outcomes measurement information system (PROMIS): depression, anxiety, and anger. *Assessment*.

[34] Choi S. W., Reise S. P., Pilkonis P. A., Hays R. D., Cella D. (2010). Efficiency of static and computer adaptive short forms compared to full-length measures of depressive symptoms. *Quality of Life Research*.

[35] Rosenberg M. (1965). *Society and the Adolescent Self-Image*.

[36] Curran C. A., Ponsford J. L., Crowe S. (2000). Coping strategies and emotional outcome following traumatic brain injury: a comparison with orthopedic patients. *Journal of Head Trauma Rehabilitation*.

[37] Anson K., Ponsford J. (2006). Evaluation of a coping skills group following traumatic brain injury. *Brain Injury*.

[38] Areán P. A., Kraemer H. C. (2013). *High Quality Psychotherapy Research: From Conception to Piloting to National Trials*.

[39] Wilder Schaaf K. P., Kreutzer J. S., Danish S. J., Pickett T. C., Rybarczyk B. D., Nichols M. G. (2013). Evaluating the needs of military and veterans' families in a polytrauma setting. *Rehabilitation Psychology*.

[40] Degeneffe C. E. (2001). Family caregiving and traumatic brain injury. *Health and Social Work*.

[41] Verhaeghe S., Defloor T., Grypdonck M. (2005). Stress and coping among families of patients with traumatic brain injury: a review of the literature. *Journal of Clinical Nursing*.

[42] Department of Veterans Affairs (2013). *VHA Handbook 1172.01: Polytrauma System of Care*.

[43] http://www.govtrack.us/congress/bills/111/s1963.

